# Factors leading to the academic failure of undergraduate medical students - Predict early to prevent

**DOI:** 10.12669/pjms.38.8.5951

**Published:** 2022

**Authors:** Azhar Rashid, Rahila Yasmeen, Rehan Ahmed, Khaulah Jawed

**Affiliations:** 1 Prof. Dr. Azhar Rashid, MBBS, FCPS, OJT, MSc (Defense & Strategic Studies), CHPE, MHPE. Dean FHMS/Principal, Islamic International Medical College, Riphah International University, Islamabad Pakistan; 2 Prof. Dr. Rahila Yasmeen, BDS, MHPE, (PhD Scholar), Dean & Head of Medical Education Dept., Riphah University, Rawalpindi, Pakistan; 3 Prof. Dr. Rehan Ahmed Khan. MBBS, FCPS, FRCS, MHPE, PhD (Medical Education), Assistant Dean Medical Education and Professor of Surgery, Islamic International Medical College, Riphah International University, Islamabad Pakistan; 4 Dr. Khaulah Jawed, BDS, MHPE. Lecturer, Department of Dental Education. Avicenna Medical and Dental College, Lahore, Pakistan

**Keywords:** Academic failure, Predict, Prevent, Factors, Undergraduate medical student

## Abstract

**Objectives:**

The main objective of the study was to explore the factors to predict academic failure before the first major assessment.

**Methods:**

An exploratory qualitative study was conducted from March 2021 to August 2021 at Riphah International University. Using the purposive sampling technique, 16 students and seven teachers were included in the study. Eight online interviews were conducted with students who were academic failures and two focus group discussions were held with eight high achievers and seven teachers. The data was analyzed and thematic analysis was done.

**Results:**

Thematic analyses deduced relevant themes which were: Educational Journey Does past matter? Essential for learning, Personality and psychological make-up, and assessment of behaviour. The factors identified were performance record, learning difficulty, educational dislocation, missionless and purposeless, against free will, tuition, poor self-regulation, low effort, procrastination, non-reflective practice, cognitive load mismanagement, limited remediation, hesitant help-seeking, low self-efficacy, introvert behaviour, demotivation, emotional imbalance, observation of student behaviour, assessment of assigned task.

**Conclusion:**

Academic failure can be predicted early and salvaged.

## INTRODUCTION

Undergraduate medical education is exacting. Stress among all the medical students is high varying between 30% and 50%^1^,^2^ but failure rate on average is 10%^3^ which means that there are other factors which lead to academic failure.

Many factors have been identified leading to academic failure, like lack of proper planning and self-regulation, psychological and personality issues, socio-demographic disposition, cognitive problems, demotivation, low self-efficacy, poor learning environment, inappropriate instructional design, teaching strategies, and others.^4^,^5^ UNESCO has defined academic failure as “to repeating the grade, early drop out and decline in the educational quality of learners”.^6^ In this study operational definition of academic failure is ‘Student failing maximum examinations, of the fourth and fifth year’.

Pinyopornpanish et al. have classified low academic achievement into three broad groups, namely personal, institutional, and social.^4^ To address the issue, institutions have developed structured selection processes and active support systems but could not prevent the scourge of academic failure.^7^,^8^

The problem is that, after failure in a major assessment, salvaging a student from future failure becomes difficult.^3^ To predict and prevent academic failure before the first major assessment is a colossal challenge. In two studies conducted on 706 and 1174 students that we found in literature, Wilkinson and Mercer found that the preadmission test and previous academic performance low scorers remain low performers in the medical school^7^,^9^ but, prior academic performance accounts for only 23% of the variance in undergraduate medical performance.^7^

The reasons mentioned demand early prediction of an academic failure. The current knowledge is scarce on the problem.^3^ Yates developed a toolkit for the detection of medical students at risk of failure in the preclinical years.^8^ and factors have been identified as well.^5^ The evidence to detect the academic failure before the first major assessment could not be identified in the literature.

This study was conducted to explore the factors which can predict a potential academic failure in undergraduate medical students before the first major assessment.

## METHODS

It is an exploratory qualitative study, conducted from March 2021 to August 2021. Ethical approval was obtained from Ethical Review Board, Riphah International University (Ref # Riphah/IRC/21/10). The study was conducted at Islamic International Medical College, where the curriculum is integrated, modular and student-centered. The first major summative assessment is ‘Combined Block Assessment (CBA)’ four months after admission into the medical college with 11 low stake/formative assessments before it.

Purposive and maximal variation sampling approach was used.^10^ Eight students, four each from fourth and fifth year failing maximum examinations since admission, eight high achievers, four top most students from fourth and fifth year with seven teachers with minimum five years of teaching experience holding diploma or master’s in health professional education (HPE), in total twenty-three were selected for in-depth interviews.

### Data Collection:

Semi-structure, open-ended questionnaires were prepared after literature search and expert validation to collect data from three groups of interviewees.^11^ Eight One to one interviews, an average of 45 minutes with the academic failures, and two focus group discussions (FGD) of 90 minutes with the eight high achievers and seven teachers were conducted on MS Teams (online).

### Data Analysis:

Thematic analysis was done. Verbatim and intelligent transcription was done manually with two cycles of coding.^12^ Reflection of the interviews and FGDs was written. Subthemes, (factors) categories, and themes were deduced and a framework was constructed from the factors.

### Quality Assurance Measures:

Credibility was ensured through member checking. Dependability was done through thick description of the methodology. Confirmability was ensured through triangulation, whereas transferability was ensured through the finest details about the context, methods, participants, and the study.^13^

## RESULTS

The factors which can predict a potential academic failure in undergraduate medical students before the first major assessment, based on the qualitative data analysis, the themes sub-theme (factors) and the relevant codes are given in Table-I.

**Table-I:** Factors responsible for academic failure.

S. No	Themes	Sub-theme (Factors)	Quotes
1	Educational journey: Does past matter?	Performance record – middle level educational dislocation - Change of medium of instruction and institution Learning difficulty Coaching, Tuition Joining profession against free will. Missionless and purposeless	AF – “I was in Pakistan. I was a good student. We went to Sharjah, there it became difficult to cope with the syllabus, as here it was in Urdu and in Sharjah it was English”.
			AF – “ I changed schools, joined Roots system, learning was very accelerated…… I had few deficiencies in Mathematics and Physics, I covered it through tuition”.
			AF – “Doctor was my father’s choice, mine was teacher or army”. “I take time to understand a topic, once I read I think I have learned but when recall it is forgotten”.
			AF – My schooling was in Riyadh, changed school in Matric. I was in first ten till class seven. I had grade ‘A’ in Matric, Grade ‘B’ in FSc”.
2	Essential for learning	Poor self-regulation Non- Reflective practice Low effort Procrastination Limited Remediation Hesitant help seekers Cognitive load mismanagement	AF – “I don’t make any plan, only plan is, follow the time table in the college but not afterwards”. AF – “I know I have done a mistake. I think on the issue but can’t do analysis”. AF – “In the first three years never asked questions from the teachers”. HA – “don’t get serious” HA – “timetable build pressure” T – “Reflection is an important component of teaching and learning, helps in understanding the core strengths and weaknesses”.
3	Personality/ Psychological make up	Low self-efficacy Introvert Demotivation (stress and fear) Emotional imbalance	AF – “When I was small had a lot of fear in myself, now only fearful of studies”. AF – “I have mood swings, my entire day and whole life depends upon emotions”. AF – “I think I can’t do anything”. HA – “not confident don’t believe in themselves”. T – “The academic failures are not motivated, have phobias anxiety, stress, depression, lack of confidence, introvert, loners, irregular….”.
4	Assessment of behaviour	Observation of student behavior Assessment of assigned task	AF – “If child is not understanding …. it is visible on the face”. AF – “Teacher can identify if a student comes unprepared by seeing the confidence or through a practical task especially in SGDs”. AF – “Low performer are not regular and remain aloof”. T– “After two formatives we can say who are low performers”. T – “Students might not do well in the first two formatives assessments, we should look at the “sum of all” that is teachers and higher administration assessment as well”
5	Social Context	Amiable social environment Communication gap	AF – “My parents did everything for me”. AF– “I was quiet and aloof as a child”.

AF = Academic failure, HA = High Achiever, T = Teacher.

## DISCUSSION

Many factors lead to academic failure. For early prediction of an academic failure literature mentions factors,^14^ and a ‘toolkit’^8^ for the preclinical years but it is deficient on the subject of prediction before the first major assessment.^15^ Our study has identified factors under four themes to predict academic failure before the first major assessment.

### Theme: Educational Journey: Does past matter?

Studies have highlighted the correlation of preadmission tests and performance. Low performers in the Australian Tertiary Admission Rank (ATAR) or Grade Point Average (GPA) remained low performers after admission into the undergraduate medical programme.^15^ Similar results are reported by other studies as well.^14^,^16^-^18^ Coaching for admission has short-term benefit.^17^ Highly structured preadmission criteria also, could not prevent selection of low performers.^8^

In our study relative low-performance record prior to the admission has been a major indicator of academic failure with antecedent reasons providing deeper insight into the issue.

### Theme: Essentials for Learning:

Poor time management and self-regulation as a major factor for poor performance has been mentioned in different studies.^5^,^19^,^20^ Pinyopornpanish et al found 79.3% low performance is due to individual factors in which absenteeism was a prominent cause.^4^ Critical thinking plays a role and hesitation for asking help adds to low performance.^14^,^20^ Our findings tally with other studies except reflective practice needs focus which was minimal in the failing students.

### Theme: Personality, Psychological Make-up:

Positive personality traits, emotional balance, motivation, and positive self-efficacy are reported as factors leading to positive outcome in academics while high-stress levels lead to negative outcome.^14^ In our study stress was a common factor in the academic failures and high achievers, difference was, former had fear with stress and for the later it was a driver. Introvert personality and low self-efficacy were other observable prominent factors contributing to academic failure. Psychological issues are not uncommon.^5^ Quarter of the low performers have psychological problems,^4^ similar to our findings.

### Theme: Assessment of Behaviour:

Behaviour and performance in the major assessments are significant factors to predict students at risk for failure in preclinical as highlighted by Yates^8^ and Ahmedy^14^ in their studies. First semester examination was a predictor in the study by James.^21^

Our finding suggests that, focused observation and assessment of the behaviour of students in the college activities and multiple formative assessments before the first CBA can predict the academic failure.

### Theme: Social Context:

Family and the institution play a role in academic performance.^20^ Negative impact of family varies, one study mentions 81.5%^6^ the other 5.2%.^4^ Our findings could not detect a significant negative impact.

### Limitation of the study:

Social context could not be explored in depth because of the limitations set by the sensitivity of the subject.

## CONCLUSION

Reasons for early detection of an academic failure before the first major assessment exist, but blinkered focus of the teachers and lack of effective instrument keeps prediction a distant possibility. Early prediction of an academic failure is vital before the first major assessment as after it the plight becomes a dilemma.

### Recommendation

- More studies to be conducted to predict factors leading to academic failure before the first major assessment.
- Framework for the prediction of academic failure before the first major assessment to be constructed for early detection of academic failure from the factors identified, leading to the development of an instrument.

### Authors’ Contribution:

**AR:** Conceptualized, data collected, conceived, designed, statistical analysis & editing.

**RY:** Reviewed and final approval of manuscript.

**RAK:** Analysis, interpretation & writing of results and editing.

**KJ:** Critical revision and editing of manuscript.

All authors are responsible and accountable for the accuracy of the work.

## References

[1] Haldorsen H, Bak NH, Dissing A, Petersson B (2014). Stress and symptoms of depression among medical students at the University of Copenhagen. Scandinavian J Public Health.

[2] Abdulghani HM (2008). Stress and depression among medical students:A cross sectional study at a medical college in Saudi Arabia. Pak J Med Sci.

[3] Bennion LD, Durning SJ, LaRochelle J, Yoon M, Schreiber-Gregory D, Reamy BV, Torre D (2018). Untying the Gordian knot:Remediation problems in medical schools that need remediation. BMC Med Educ.

[4] Pinyopornpanish M, Sribanditmongkok P, Boonyanaruthee V, Chan-ob T, Maneetorn N, Uuphanthsath R (2004). Factors affecting low academic achievement of medical students in the faculty of medicine, Chiang Mai University. Changi Mai Med Bull.

[5] Kiran F, Javaid A (2020). Students'perceptions of factors for academic failure in pre-clinical years of a medical school. J Pak Med Assoc.

[6] Najimi A, Sharifirad G, Amini M, Meftagh S (2013). Academic failure and students′viewpoint:The influence of individual, internal and external organizational factors. J Educ Health Promot.

[7] Wilkinson D, Zhang J, Byrne GJ, Luke H, Ozolins IZ, Parker MH, Peterson RF (2008). Medical school selection criteria and the prediction of academic performance. Med J Aus.

[8] Yates J (2011). Development of a 'toolkit'to identify medical students at risk of failure to thrive on the course:an exploratory retrospective case study. BMC Med Educ.

[9] Mercer A, Puddey IB (2011). Admission selection criteria as predictors of outcomes in an undergraduate medical course:A prospective study. Med Teach.

[10] Creswell JW (2012). Educational Research Planning, Conducting and Evaluating Quantitative and Qualitative Research - 4th edition, Pearson Education Inc.

[11] Artino AR, La Rochelle JS, Dezee KJ, Gehlbach H (2014). Developing questionnaires for educational research:AMEE Guide No. 87. Med Teach.

[12] Saldana J, Jai Seaman The Coding Manual for Qualitative Researchers (3rd edition). Second. Vol. 12, Qualitative Research in Organizations and Management:An International Journal.

[13] Korstjens I, Moser A (2018). Series:Practical guidance to qualitative research. Part 4:Trustworthiness and publishing. Eur J Gen Pract.

[14] Ahmady S, Khajeali N, Sharifi F, Mirmoghtadaei ZS (2019). Factors related to academic failure in preclinical medical education:A systematic review. J Adv Med Educ Prof.

[15] Li J, Thompson R, Shulruf B (2019). Struggling with strugglers:Using data from selection tools for early identification of medical students at risk of failure. BMC Med Educ.

[16] Yousafzai II, Jamil B (2019). Relationship between admission criteria and academic performance:A correlational study in nursing students. Pak J Med Sci.

[17] Griffin B, Bayl-Smith P, Hu W (2018). Predicting patterns of change and stability in student performance across a medical degree. Med Educ.

[18] Simpson PL, Scicluna HA, Jones PD, Cole A, O'Sullivan AJ, Harris PG (2014). Predictive validity of a new integrated selection process for medical school admission. BMC Med Educ.

[19] Patel R, Tarrant C, Bonas S, Yates J, Sandars J (2015). The struggling student:a thematic analysis from the self-regulated learning perspective. Med Educ.

[20] Malau-Aduli BS, Ray RA, O'connor T, van der Kruk Y, Alele FO, Bellingan M (2020). Dealing with academic difficulty in medical school:A pilot study. Educ Sci.

[21] James D, Yates J, Ferguson E (2013). Can the 12-item general health questionnaire be used to identify medical students who might 'struggle'on the medical course?A prospective study on two cohorts. BMC Med Educ.

